# The Role of Sublobar Resection in Early-Stage Non-Small-Cell Lung Cancer

**DOI:** 10.3390/jcm13175277

**Published:** 2024-09-05

**Authors:** Francesco Petrella, Andrea Cara, Enrico Mario Cassina, Sara Degiovanni, Lidia Libretti, Emanuele Pirondini, Federico Raveglia, Antonio Tuoro, Sara Vaquer

**Affiliations:** Department of Thoracic Surgery, Fondazione IRCCS San Gerardo dei Tintori, 20900 Monza, Italy; andrea.cara@irccs-sangerardo.it (A.C.); enricomario.cassina@irccs-sangerardo.it (E.M.C.); sara.degiovanni@unimi.it.it (S.D.); lidia.libretti@irccs-sangerardo.it (L.L.); emanuele.pirondini@irccs-sangerardo.it (E.P.); federico.raveglia@irccs-sangerardo.it (F.R.); antonio.tuoro@irccs-sangerardo.it (A.T.); sara.vaquer@unimi.it (S.V.)

**Keywords:** lobectomy, segmentecomy, wedge resection, randomized controlled trial, overall survival, disease free survival

## Abstract

The results of a prospective, multi-institutional randomized trial developed to assess the equality of sublobar resection versus standard lobectomy were first published in 1995. They concluded that, compared with lobectomy, sublobar resections did not show any significant improvement either in terms of postoperative morbidity and mortality nor in terms of late post-resectional cardiorespiratory function. Moreover, due to the higher mortality and local recurrence rate related to sublobar resection, lobectomy had to be judged as the best surgical option for patients diagnosed with peripheral early-stage non-small-cell lung cancer. Since then, lobectomy has been considered the best surgical option for fit patients suffering from early-stage non-small cell lung cancer. In 2022 and 2023, three non-inferiority randomized trials were published, comparing lobectomy with the sublobar resection in T1a N0 patients whose tumors were up to 2 cm in size. Although presenting some important differences, all three trials met their primary endpoints, disclosing the non-inferiority of sublobar resections in terms of overall and disease-free survival. This narrative review aims to compare the newly published results of these trials as well as to report results from recent non-randomized studies on this topic.

## 1. Introduction

On 5 April 1933, at the Barnes Hospital in St Louis, Evarts A. Graham—assisted in the operation by William D. Adams—realized the first successful lung resection for lung cancer by way of a pneumonectomy on a 48-year-old obstetrician from Pittsburgh, James L. Gilmore, who survived for a long time and outlived Graham, who himself died of pulmonary neoplasm in 1957 [1]. In 1939, Churchill and Belsey—from the thoracic clinic and surgical services of the Massachusetts General Hospital in Boston—performed the first pulmonary segmentectomy—a lingulectomy—for bronchiectasis, a clinical and pathological entity first described by the French physician René-Théophile-Hyacinthe Laënnec as a temporary dilatation of the bronchi caused by voluminous sputum, subsequently made permanent by continuous secretion [2,3].

More than twenty years later, in 1962, Shimkin et al. from the National Cancer Institute in Bethesda, comparing the impact of lobectomy and pneumonectomy on lung cancer patients—using the data from Alton Ochsner of New Orleans and Richard H. Overholt of Boston–demonstrated that less extended pulmonary resections provided survival rates that were at least as good as, and perhaps better than, those reported after more aggressive operations [4]. Since then, pulmonary lobectomy became the gold-standard surgical treatment for lung cancer, and segmentectomy was offered only to less fit patients not amenable to major resection. In 1973, Jensik et al. from the Department of Cardiovascular Thoracic Surgery, Rush-Presbyterian-St. Luke’s Medical Center in Chicago described a fifteen-year experience of segmental resection for cancer in 119 selected patients receiving 123 resections with a mortality rate of 4.8%; lung segmentectomy was indicated in the case of the following: history of an already performed contralateral resection; the necessity for a palliative resection and for resection of a peripheral lesion. They reported a 5-year survival rate for the patients receiving radical resection of 56.4% [5].

In 1994, Warren and Faber, from the Department of Cardiovascular-Thoracic Surgery of the Rush-Presbyterian-St. Luke’s Medical Center in Chicago reported five-year survival rates and patterns of intrathoracic recurrence of segmentectomy versus lobectomy in patients with stage I pulmonary carcinoma. Although this study did not disclose any benefit of lobectomy over segmental resection—in terms of survival—for patients diagnosed with tumors of 3.0 cm or smaller, a survival advantage was observed for patients receiving lobectomy for neoplasms larger than 3.0 cm. The local recurrence rate was significantly higher for segmental resection: 22.7% after sub anatomical resection versus 4.9% after lobectomy. The authors then concluded that lobectomy was the best surgical resection for NSCLC stage I patients presenting tumors larger than 3.0 cm. On the contrary, in tumors less than 3 cm, no survival advantage was reported [6].

In 1995, Ginsberg et al. published the results of the LCSG 0821 trial, a prospective, multi-institutional randomized trial developed to assess the equivalence of sublobar resection versus standard lobectomy. They concluded that, compared with lobectomy, sublobar resections did not show any significant improvement either in terms of postoperative morbidity and mortality or in terms of late postoperative cardiorespiratory performance. Moreover, due to the higher mortality and local recurrence rate related to sublobar resection, lobectomy had to be considered the best surgical option for patients diagnosed with peripherally located T1N0 NSCLC [7].

Since then, lobectomy has been judged as the best surgical option for fit patients suffering from early-stage NSCLC; on the other hand, sublobar resections were indicated for patients with impaired cardiopulmonary function. In 2022 and 2023, the Japanese Clinical Oncology Group (JCOG) and the Cancer and Leukemia Group B (CALGB) co-operative group (now a part of the Alliance for Clinical Trials in Oncology) and the Deutsche Register Klinischer Studien started three non-inferiority randomized trials to compare lobectomy with sublobar resection in T1a N0 patients, whose tumor was up to 2 cm in size. Although presenting some important differences in terms of design, demographics, and patient outcomes, all three trials met their primary endpoints, disclosing the non-inferiority of sublobar resections in terms of overall and disease-free survival when compared to standard lobectomy [8].

## 2. Lobectomy, Segmentectomy, Wedge Resection: Surgical Anatomy and Technical Details

Normal pulmonary anatomy accounts for three lobes on the right side (upper, middle, and lower) and two lobes on the left side (upper and lower). Each lobe is subdivided by bronchopulmonary segments which are as follows: in the right upper lobe, the apical segment (B1), posterior segment (B2), and anterior segment (B3) are present; in the middle lobe, the lateral segment (B4) and medial segment (B5) are present; and in the right lower lobe, the superior segment (B6), medial segment (B7), anterior segment (B8), lateral segment (B9), and posterior segment (B10) are present. In the left upper lobe, the apico-posterior segment (B1/2), anterior segment (B3), superior lingular segment (B4), and inferior lingular segment (B5) are present; in the left lower lobe, the superior segment (B6), antero-medial segment (B7/8), lateral segment (B9), and posterior segment (B10) are present. In light of this classification, there are 10 segments in the right lung, while in the left lung—depending on how the apico-posterior of the upper lobe and the antero-medial of the lower lobe are classified—eight to ten segments are present. Each segment has its own broncho-vascular supply, and it is anatomically and functionally autonomous, thus permitting a single-segment resection without affecting its neighboring segments.

Lobectomy is an anatomical resection requiring the division of the bronchus of the lobe to be resected, including pulmonary artery branches and pulmonary venous drainage to each lobe individually. On the other hand, anatomic segmentectomy—also defined as typical segmentecomy—requires the division of the segmental bronchus, artery, and vein. Wedge resection—also defined as atypical segmentectomy—is a non-anatomical resection—which does not require individual division of hilar structures—allowing peripheral nodule excision, surrounded by a margin of normal lung, via trans-segmental resection.

## 3. Non-Randomized Studies

In 1997, Kodama et al. [9] reviewed their experience comparing standard lobectomy and limited resection for early-stage NSCLC using concurrent non-randomized controls. They analyzed data from 63 patients, among which 46 patients received a segmentectomy as an intentional treatment, and the remaining 17 received treatment because of impaired pulmonary function. The 5-year survival rate was 93% in the planned resection group and was not different from patients undergoing standard lobectomy and complete mediastinal lymph node dissection. On the basis of their results, the authors concluded that segmentectomy with complete hilar and mediastinal dissection should be considered as an appropriate alternative therapeutic option for selected patients with early-stage NSCLC [9].

In 2006, Okada et al. [10] published the results of a non-randomized study performed between 1992 and 2001 at three institutes comparing standard lobectomy with sublobar resection (segmentectomy or wedge resection) for patients affected by peripheral cT1N0M0 non-small-cell lung cancer of 2 cm or less in major diameter.

Both groups disclosed similar disease-free and overall survivals, and multivariate analysis confirmed that sublobar resections were not inferior to lobectomy both in terms of recurrence rate and global prognosis. The authors concluded their paper by recommending randomized controlled trials to confirm the non-inferiority of sublobar resection versus standard lobectomy in peripherally located early-stage NSCLC with a major diameter of less than two [10].

In 2011, Whitson et al. [11] published their study using the Surveillance Epidemiology and End Results (SEERs) database from 1998 to 2007. They focused on patients receiving anatomic segmentectomy or standard lobectomy for stage I adenocarcinoma or squamous cell carcinoma; wedge resections were not enrolled in the analysis. They stratified the patients based on neoplasm diameter and then analyzed the association between the extent of resection and survival. The authors observed that lobectomy disclosed superior unadjusted overall and cancer-specific 5-year survival compared with segmentectomy; moreover, after adjusting for patient and tumor factors, standard lobectomy was confirmed to provide better overall and cancer-specific survival rates, regardless of tumor size. On the basis of their results, the authors concluded that standard lobectomy was shown to confer a significant survival benefit when compared with segmentectomy, thus confirming the role of lobectomy as the standard surgical treatment of stage I NSCLC [11].

In 2015, Khullar et al. [12] published a retrospective cohort study utilizing the National Cancer Data Base. They evaluated patients receiving lobectomy, segmentectomy, or wedge resection for early-stage NSCLC. A total of 987 patients were enrolled in each arm. Both sub anatomic resections disclosed worse overall survival when compared with standard lobectomy, although no difference was reported in terms of 30-day mortality. In fact, the median overall survival rates were 100, 74, and 68 months for lobectomy, segmentectomy, and wedge resection, respectively; in addition, sub anatomic resection was related to a higher probability of positive resection margins, a lower chance of having more than three lymph nodes analyzed, and lower rates of lymph nodal upstaging. On the basis of their results, the authors concluded that both wedge and segmental resections disclosed significantly worse overall survival when compared to standard lobectomy [12].

In 2016, Spiecher et al. published an analysis of the National Cancer Data Base from 2003 to 2011 focusing on variables associated with the practice of sublobar resection versus standard lobectomy for treating early-stage NSCLC. Among the 39,403 patients considered for analysis, 29,736 (75.5%) were submitted to standard lobectomy, and 9667 (24.5%) received sub anatomic resection; among the patients receiving sub anatomic resection, 84.7% received wedge resection (8192 patients), and 15.3% received anatomical segmentectomy (1475 patients). Overall, standard lobectomy disclosed a significantly higher 5-year survival compared to limited resection. Also, in the sublobar resection arm, nodal sampling disclosed a significantly better 5-year survival [13].

In 2018, Subramanian et al. compared standard lobectomy with sub anatomical resections using data from the National Cancer Data Base, identifying 1687 patients diagnosed with stage IA NSCLC undergoing lobectomy (1354) and sublobar resection (333). Although standard lobectomy and the sub anatomic resection arm had similar 5-year overall survival rates (61.8% vs. 55.6%, *p* = 0.561), the sublobar arm showed a 39% increased risk of NSCLC recurrence [14].

## 4. The Lung Cancer Study Group Trial

In 1995, Ginsberg et al. published the results of a prospective, multi-institutional randomized trial comparing sub anatomical resection with standard pulmonary lobectomy for patients diagnosed with peripheral T1 N0 NSCLC. The study started in February 1982 and ended in November 1988, enrolling 247 patients, 122 undergoing limited resection and 125 undergoing lobectomy. The arm of limited resections included both segmental resections and wedge resections: segmental resection required isolation, division, and suture of the appropriate segmental bronchus, artery, and vein. Large wedge resections with proper resection margins—with at least 2 cm of non-involved lung tissue—were considered. All required lymph node stations—draining the affected pulmonary segment—were sampled, and frozen sections had to confirm the absence of nodal involvement, thus proving the pathological N0 disease. In the case of incomplete resection or more advanced intraoperative findings than the T1N0 stage, lobectomy had to be performed. No significant differences were reported in terms of postoperative morbidity and mortality, although in the standard lobectomy arm, six patients required postoperative ventilation lasting more than 24 h because of postoperative acute respiratory failure. During the postoperative period, three patients died, one in the sublobar group and two in the standard lobectomy group.

In terms of survival difference, both the overall and the oncologic death rates were lower after lobectomy than after sublobar resection; in detail, sublobar resections were shown to have a 30% rise in the overall death rate. Moreover, they presented a 50% rise in the oncologic death rate. An increase in the recurrence rate of 50% was observed in the sublobar group, regardless of whether the intended operation was wedge resection or segmentectomy. Analyzing the recurrence rate by site, the sublobar resection group disclosed the tripling of the locoregional recurrence rate, while the distant recurrence rate was not affected by surgical treatment.

With regard to the preservation of pulmonary function, a 6-month follow-up spirometric assessment disclosed a clear advantage of sublobar resection, which was no longer observed in 12- and 18-month pulmonary function tests.

In conclusion, although the study was designed to prove that less extended resection would offer similar results—in terms of cancer-free survival and local recurrence—when compared to standard lobectomy, the results of the trial disclosed that sublobar resection had an increased chance of local recurrence and a reduced probability of overall survival and disease-free survival [7].

## 5. The JCOG0802/WJOG4607L Trial

In 2022, Saji et al. published a multi-center, open-label, phase 3, randomized, controlled, non-inferiority trial comparing segmentectomy versus lobectomy in peripherally located NSCLC with small size. The trial was conducted between August 2009 and October 2014 at 70 institutions in Japan, enrolling 1106 patients who received either lobectomy (554 patients) or segmentectomy (552 patients). Indications were fit patients (with a performance score of 0 or 1) with peripheral NSCLC smaller than 2 cm in diameter, located in the outer one-third of the lung, and with a consolidation-to-tumor ratio > 0.5, resulting in clinical stage IA. In the standard lobectomy cohort, lobectomy was performed, and mediastinal and hilar lymph node dissections were routinely conducted. In the segmentectomy group, segmentectomy was defined as the resection of only one segment or with an additional adjacent segment; hilar and mediastinal lymph node dissections were routinely performed. In both the standard lobectomy and segmentecomy groups, systematic or selective lymphadenectomy was accomplished; whenever frozen sections diagnosed nodal metastases, a modification in the surgical strategy was applied, if necessary, to achieve complete resection. The primary endpoint was overall survival; among others, the spirometric assessment of postoperative respiratory performance at 6 and 12 months was one of the secondary endpoints. Segmentectomy disclosed a 5-year overall survival of 94.3% and lobectomy of 91.1, thus confirming the non-inferiority of segmentectomy; the difference in the reduction in median forced expiratory volume for 1 s (FEV 1) between the two groups—measured 1 year after surgery—was only 3.5%, thus not reaching the pre-assumed value for a clinical impact of 10%. In this trial, the incidence of death from other diseases after lobectomy was higher, and this has been attributed to the non-inferiority of sublobar resections in terms of overall survival.

The authors concluded that lobectomy—in the case of small-sized, peripheral cT1N0 NSCLC—might represent an overtreatment—on the basis of long-term survival—and they propose segmentectomy as the new standard surgical therapy for small-sized peripheral stage IA NSCLC [15].

## 6. The Deutsche Register Klinischer Studien DRKS00004897 Trial

In 2022, Stamatis et al. [13] published the results of a prospective, randomized, Phase-III multi-center study carried out at 11 centers in Germany, Switzerland, and Austria from October 2013 to June 2021. The trial aimed to show the non-inferiority of segmentectomy versus lobectomy, and the primary endpoint was 5-year overall survival in patients diagnosed with NSCLC smaller than 2 cm in diameter and no lymph nodal metastases. The surgical approach (thoracotomy versus video-assisted surgery) was at the surgeon’s discretion. Anatomical segmentectomy was conducted by the dissection, isolation, and suture of each structure of the segmental hilum (artery, vein, and bronchus); inflation of the parenchyma was used to correctly identify the intersegmental plane. Lobectomy was performed in a standard way by an open or minimally invasive approach. In the case of segmentectomy, frozen sections were required for the intraoperative assessment of resection margins and lymph node status; if parenchymal resection margins were less than 2 cm, an extension of the resection to the closest segment was performed; if regional lymph nodes were positive, standard lobectomy was performed.

Intraoperative and 90-day mortality were 0% in both groups, while in-hospital morbidity was 13.2% in the segmentectomy cohort and 13.0% in the lobectomy cohort. No significant differences were observed in the two groups in terms of operative time, intraoperative blood loss, and the volume of drainage secretion in the early postoperative period. No significant differences were reported in the two arms in terms of loco-regional or distant recurrences.

The lobectomy arm disclosed an overall survival of 86.52%, while the segmentectomy arm showed an overall survival at 5 years of 78.21%. Disease-free survival at 5 years was 77.29% for the lobectomy group and 79.96% for the segmentectomy group, although targeted non-inferiority was shown only for disease-free survival after multivariable adjustment. The authors concluded that—as the non-inferiority of segmentectomy was definitively confirmed only for disease-free survival but not for overall survival—their preliminary results need to be proven by further and larger randomized studies [16].

## 7. The CALGB 140503 Trial

In 2023, Altorki et al. [17] published a multi-center, non-inferiority, phase 3 trial comparing sublobar resection vs. standard lobectomy for the surgical treatment of stage T1aN0 NSCLC with a tumor size ≤2 cm in diameter. The trial was conducted between June 2007 and March 2017 at 83 institutions in the United States, Australia, and Canada, enrolling 697 patients who received either lobectomy (357 patients) or sublobar resection (340 patients). Among the 340 patients who received sublobar resection, 201 (59.1%) were submitted to wedge resection, and 129 (37.9%) received an anatomical segmental resection. Inclusion criteria were the presence of a peripheral pulmonary nodule—presenting at least a clear solid part—2 cm in diameter or less, located in the outer third of the lung, which would, thus, be amenable to sublobar resection or standard lobectomy, with a performance-status score of 0, 1, or 2. Patients presenting pure ground glass opacities (GGOs) or pathological N1 or N2 lymph node involvement were excluded from the trial. Once intraoperative eligibility criteria were satisfied, patients were randomized 1:1 to either sub anatomic resection or lobectomy. The surgeon decided the type of surgical approach (open versus minimally invasive video-assisted or robotic procedure) and—in the case of sublobar resection—the type of resection (wedge resection or segmentectomy). Disease-free survival was the primary endpoint, while overall survival, systemic or local recurrences, and FEV1 six months after surgical resection were secondary endpoints.

The 5-year disease-free survival was 63.6% for sublobar resection and 64.1% for standard lobectomy, thus disclosing that sublobar resection is not inferior to lobectomy for disease-free survival. The 5-year overall survival was 80.3% after sublobar resection and 78.9% after lobectomy, thus disclosing similar results for the two groups. Disease recurrence was observed in 30.4% of patients in the sublobar resection group and in 29.3% of patients in the standard lobectomy group. Locoregional recurrence was reported in 13.4% of patients in the sublobar resection group and in 10% of patients in the standard lobectomy group. The 5-year recurrence-free survival was 70.2% after sub anatomic resection and 71.2% after lobar resection. Six months after the procedure, a greater decrease in FEV1 was observed in the lobectomy group (−6.0) than after sublobar resection (−4.0). On the basis of their results, the authors concluded that sublobar resections (wedge resection or segmentectomy) in patients with early-stage NSCLC are effective therapeutic options with similar results to standard lobectomy [17].

## 8. JCOG0802/WJOG4607L vs. CALGB 140503 vs. DRKS00004897

All three randomized control trials reached the same conclusion, suggesting the non-inferiority of segmentecomy when compared to standard lobectomy, although this varied for several major characteristics and endpoints, such as trial design, enrollment criteria, technical details, and outcomes. All three studies were phase III non-inferiority trials, whose primary endpoints were disease-free survival and overall survival, and all three trials provided level I evidence of the non-inferiority of sublobar resection versus standard lobectomy in terms of long-term survival in patients diagnosed with small peripheral early-stage NSCLC. However, several significant differences were also highlighted: first, both DRKS00004897 and JCOG0802 included only segmentecomy in the sublobar resection arm, not allowing for wedge resection; on the other hand, CALGB 140503 enrolled both segmentectomy and wedge resections in the sublobar resection arm. Moreover, regarding imaging enrollment criteria, JCOG0802 was more restrictive, enrolling only lesions smaller than 2 cm in diameter and a consolidation tumor ratio greater than 0.5 on preoperative thin-section computed tomography; on the other hand, the CALGB 140503 trial enrolled only peripheral nodules with a diameter less than 2 cm localized in the external part of the lung, without any requirement regarding consolidation tumor ratio; and finally, the DRKS00004897 trial was the least restrictive, requiring only that the size criteria of the nodule be less than 2 cm.

Although all three trials required histologic lung cancer diagnosis preoperatively or intraoperatively via a frozen section examination, the CALGB 140503 trial was the only one to assess the pathologic confirmation of lymph nodes’ non-involvement before randomization, thus restricting the study to true pathologic stage IA1 and stage IA2.

With regard to the intraoperative margin assessment, the three trials showed some analogies and several technical differences: the CALGB 140503 trial converted the wedge or segmentectomy to option lobectomy in the case of positive intraoperative margins. In the JCOG0802 trial, frozen sections were required during segmentectomy only when resection margins were judged to be less than the maximum tumor diameter or less than 2 cm at macroscopic examination; moreover, frozen sections were performed in the case of suspected lymph node metastatic involvement and—in the case of confirmation—standard lobectomy was performed. The DRKS00004897 trial required frozen sections—in the case of segmentectomy—both for parenchymal resection margins and lymph node assessment; in the case of parenchymal margin infiltration, further partial or total resection of the closer segment was required. In the case of lymph node metastasis, an extension to completion lobectomy was performed; however, in this trial, an adequate resection margin was always confirmed.

Both JCOG0802 and DRKS00004897 had overall survival as the primary endpoint, whereas disease-free survival was the primary endpoint for CALGB 140503. None of the three trials disclosed any significant difference in survival comparing sublobar resection and lobectomy; moreover, while both JCOG0802 and CALGB reported a significantly higher locoregional recurrence rate in the sub anatomical resection arm when compared to the standard lobectomy arm, DRKS00004897 did not confirm any significant difference between the two cohorts of patients.

Both CALGB 140503 and DRKS00004897 disclosed no difference between sublobar and lobar resections in terms of overall survival, thus confirming the non-inferiority of sub anatomical resection versus lobectomy in the study populations; interestingly, JCOG0802 disclosed a moderately better overall survival after segmentectomy, although the study was not designed and powered for superiority.

The secondary endpoint was the pulmonary function decrease after surgery by spirometric assessment: both CALGB 140503 and JCOG0802 trials disclosed a significant difference in respiratory function 6 months after resection; however, whether this spirometric decrease is clinically relevant is still unclear, as previously reported in several studies concerning staged metastasectomy [18]. Both clinical and pathologic preoperative lymph node assessments remain pivotal tools before NSCLC resection [19,20], although all three randomized trials relied heavily on intraoperative frozen section for the definitive surgical strategy (Table 1).

## 9. Conclusions

Since the results of the first randomized trial in 1995 comparing sublobar resection with lobectomy, lobectomy has been judged as the best surgical option for fit patients suffering from early-stage NSCLC; on the other hand, sublobar resections have been indicated for patients with impaired cardiopulmonary function. Three more recent randomized non-inferiority trials (2022 and 2023) have successfully demonstrated that sublobar resections (wedge resection or segmentectomy) in patients with early-stage NSCLC are effective therapeutic options with similar results to standard lobectomy. Although several non-randomized studies suggest better oncologic results from lobectomy rather than sublobar resection, this might be attributed to selection bias, as sublobar resection has been usually performed in less fit or compromised patients in non-randomized trials.

## Figures and Tables

**Table 1:** JCOG0802/WJOG4607L vs. CALGB 140503 vs. DRKS00004897.

	JCOG0802/WJOG4607L	CALGB 140503	DRKS00004897
Study design	Phase III non-inferiority	Phase III non-inferiority	Phase III non-inferiority
Sublobar resection	Segmentectomy	Wedge resection Segmentectomy	Segmentectomy
Primary endpoints	Overall survival	Disease-free survival	Overall survival
Secondary endpoints	Significant difference in respiratory function 6 months after resection *	Significant difference in respiratory function 6 months after resection *	Not reported
Enrollment criteria	Nodule < 2cm consolidation/tumor ratio > 0.5	Peripheral nodule diameter < 2 cm External part of the lung Consolidation/tumor ratio: none	Nodule < 2 cm
Pathologic confirmation of lymph nodes’ non-involvement before randomization	No	Yes	No
Frozen section	Required only ifsmacroscopic margin infiltration was suspected Suspected lymph node involvement	Conversion to lobectomy if margin positive	An adequate resection margin was always confirmed
Locoregional recurrence rate	Significantly higher locoregional recurrence rate in the sub anatomical resection arm	Significantly higher locoregional recurrence rate in the sub anatomical resection arm	Non-significant difference
Overall survival	Moderately better after segmentectomy **	No difference between sublobar and lobar resections	No difference between sublobar and lobar resections

*—not clinically relevant; **—not powered for superiority.

## Data Availability

All data are available upon request.
